# An Innovative Technique to Assess Spontaneous Baroreflex Sensitivity with Short Data Segments: Multiple Trigonometric Regressive Spectral Analysis

**DOI:** 10.3389/fphys.2018.00010

**Published:** 2018-01-22

**Authors:** Kai Li, Heinz Rüdiger, Rocco Haase, Tjalf Ziemssen

**Affiliations:** ^1^Autonomic and Neuroendocrinological Lab, Department of Neurology, Center of Clinical Neuroscience, University Hospital Carl-Gustav Carus, Technical University of Dresden, Dresden, Germany; ^2^Department of Neurology, Beijing Hospital, National Center of Gerontology, Beijing, China

**Keywords:** baroreflex sensitivity, multiple trigonometric regressive spectral analysis, baroreflex function, data segment, autonomic nervous system

## Abstract

**Objective:** As the multiple trigonometric regressive spectral (MTRS) analysis is extraordinary in its ability to analyze short local data segments down to 12 s, we wanted to evaluate the impact of the data segment settings by applying the technique of MTRS analysis for baroreflex sensitivity (BRS) estimation using a standardized data pool.

**Methods:** Spectral and baroreflex analyses were performed on the EuroBaVar dataset (42 recordings, including lying and standing positions). For this analysis, the technique of MTRS was used. We used different global and local data segment lengths, and chose the global data segments from different positions. Three global data segments of 1 and 2 min and three local data segments of 12, 20, and 30 s were used in MTRS analysis for BRS.

**Results:** All the BRS-values calculated on the three global data segments were highly correlated, both in the supine and standing positions; the different global data segments provided similar BRS estimations. When using different local data segments, all the BRS-values were also highly correlated. However, in the supine position, using short local data segments of 12 s overestimated BRS compared with those using 20 and 30 s. In the standing position, the BRS estimations using different local data segments were comparable. There was no proportional bias for the comparisons between different BRS estimations.

**Conclusion:** We demonstrate that BRS estimation by the MTRS technique is stable when using different global data segments, and MTRS is extraordinary in its ability to evaluate BRS in even short local data segments (20 and 30 s). Because of the non-stationary character of most biosignals, the MTRS technique would be preferable for BRS analysis especially in conditions when only short stationary data segments are available or when dynamic changes of BRS should be monitored.

## Introduction

The arterial baroreflex is of fundamental importance for cardiovascular homeostasis, and its impairment may play an adverse role in several diseases (Ziemssen et al., 2013). There is an inverse relation between baroreflex sensitivity (BRS) and the risk of mortality after myocardial infarction, and interventions that improve BRS had beneficial clinical impact on cardiovascular mortality (La Rovere et al., 1988, 2002; La Rovere, 2000).

Numerous non-invasive techniques that analyze the sensitivity of spontaneous baroreflex control of heart rate and blood pressure (BP) have been developed (Bertinieri et al., 1985; Blaber et al., 1995; Ducher et al., 1995; Di Rienzo et al., 2001; Laude et al., 2004; Westerhof et al., 2004; Bernardi et al., 2010; Porta et al., 2013; Svacinova et al., 2015). They differ in their general approaches (analysis in time, frequency, or information domains; causal vs. non-causal analyses) and the applied statistical details (length of data segment analyzed, window technique, number of single BRS-values for analysis). Because of their considerable differences, studies comparing the performance of these methods are needed.

The EuroBaVar study compared 21 methods of BRS analysis (Laude et al., 2004). The multiple trigonometric regressive spectral analysis (MTRS) technique for BRS evaluation had an excellent performance in comparison with the sequence methods and the spectral analysis based on fast Fourier transform. In addition, due to the short local data segments, MTRS is able to analyze BRS in short dynamic ECG and BP recordings.

BRS-values are non-stationary and profoundly variable. We therefore shorten the window for BRS analysis. For the MTRS technique, there are two relevant types of data segments, the local and the global data segments. Each trigonometric regressive spectral analysis (TRS) spectrum is only performed within a local data segment; analyses of local data segments are repeated in successive segments shifted by one, two, or more beats within the whole global data segment (multiple TRS analysis, so called MTRS) (Rüdiger et al., 1999; Ziemssen et al., 2013). In a local data segment of 25 s, even the longest oscillation of the low frequency (LF) band can be reliably determined, but additional longer oscillations could not be accurately detected. Certainly, oscillations in the high frequency (HF) band can be well characterized in this time window. However, because of their short wavelengths, oscillations in the HF band or ultra-high frequency (UHF) band can slightly change in frequency and amplitude using a local data segment of 25 s. Therefore, there is always a compromise in the selection of the local data segment length. We have applied different lengths of local and global data segments according to the research conditions (Wright et al., 2009; Friedrich et al., 2010; Reimann et al., 2010, 2012, 2013; Gasch et al., 2011; Viehweg et al., 2016; Li et al., 2017). The most commonly used lengths of local and global data segments were 30 s and 2 min, respectively, but shorter lengths have also been applied. Until now, the effect of the lengths of local and global data segments on the BRS estimation remains unclear, and the reproducibility of selecting different global data segments in the same recording is unknown. In the present study, we aimed to investigate the influence of different local and global data segment settings on BRS assessment using the EuroBaVar dataset, which is comprised of a heterogeneous population.

## Subjects and methods

### The EuroBaVar dataset

At first, this dataset was used as part of the EuroBaVar study to compare estimates of the BRS obtained by different research laboratories, each using its own software (Laude et al., 2004). The dataset included 46 recording files obtained from a heterogeneous population of 21 subjects. There are four duplicate recording files from two subjects (two lying recordings and two standing recordings). Thus a total of 42 recordings were analyzed in the present study. The participants (17 women, 4 men) were composed of 12 normotensive outpatients (including one diabetic patient without cardiac neuropathy, 2 treated patients with hypercholesterolemia, and one 3-month pregnant woman), one untreated hypertensive patient, two treated patients with hypertension, four healthy volunteers, and two patients with evident cardiac autonomic failure (one patient with diabetic neuropathy and the other patient recently underwent heart transplantation). All the participants underwent continuous non-invasive BP monitoring and ECG recording. These recordings were made for 10–12 min both in the supine position and in the upright position. Data were provided as the BP and ECG signals sampled at 500 Hz with a 16-bit resolution. This is a heterogeneous population characterized by a large range of BRS-values, and can thoroughly evaluate the performance of different BRS calculation methods. This challenging dataset is appropriate for measuring the consistency of BRS obtained by MTRS using different local and global data segment settings. The EuroBaVar data are from the EuroBaVar study, and publicly available for methodological studies on BRS analysis. The study was approved by the Paris-Necker committee and all the subjects had given informed consent.

### The MTRS analysis

In 1999, Rüdiger and colleagues introduced TRS, which detects true, physiological oscillations and guarantees an optimal assessment of the measured RR intervals and other parameters (Rüdiger et al., 1999). All oscillations are captured based on the following condition∑ (RRI(t(i)) − Reg(t(i)))^2^ => minimum, with RRI(t(i)) being the original RR intervals and Reg(t(i)) = A ^*^ sin (ωt(i) + φ(i)) being a trigonometric function of the parameters A (amplitude), ω (frequency), and ϕ (phase shift). This trigonometric function cannot be solved as none of the three parameters in the regressive function is known. Therefore, it is necessary to set one parameter, in general the frequency ω. By the variation of this frequency ω, oscillations can be calculated with an optimal variance reduction for all target frequencies.

### Computation of BRS using MTRS

The baroreflex provides a rapid feedback loop to keep cardiovascular homeostasis. For example, an increased BP reflexively leads to a decrease of the heart rate in order to keep the homeostasis of BP. This feedback loop works continuously during the constant fluctuations of heart rate and BP. This is the theoretical foundation of MTRS based BRS measurement.

**Figure 1:**
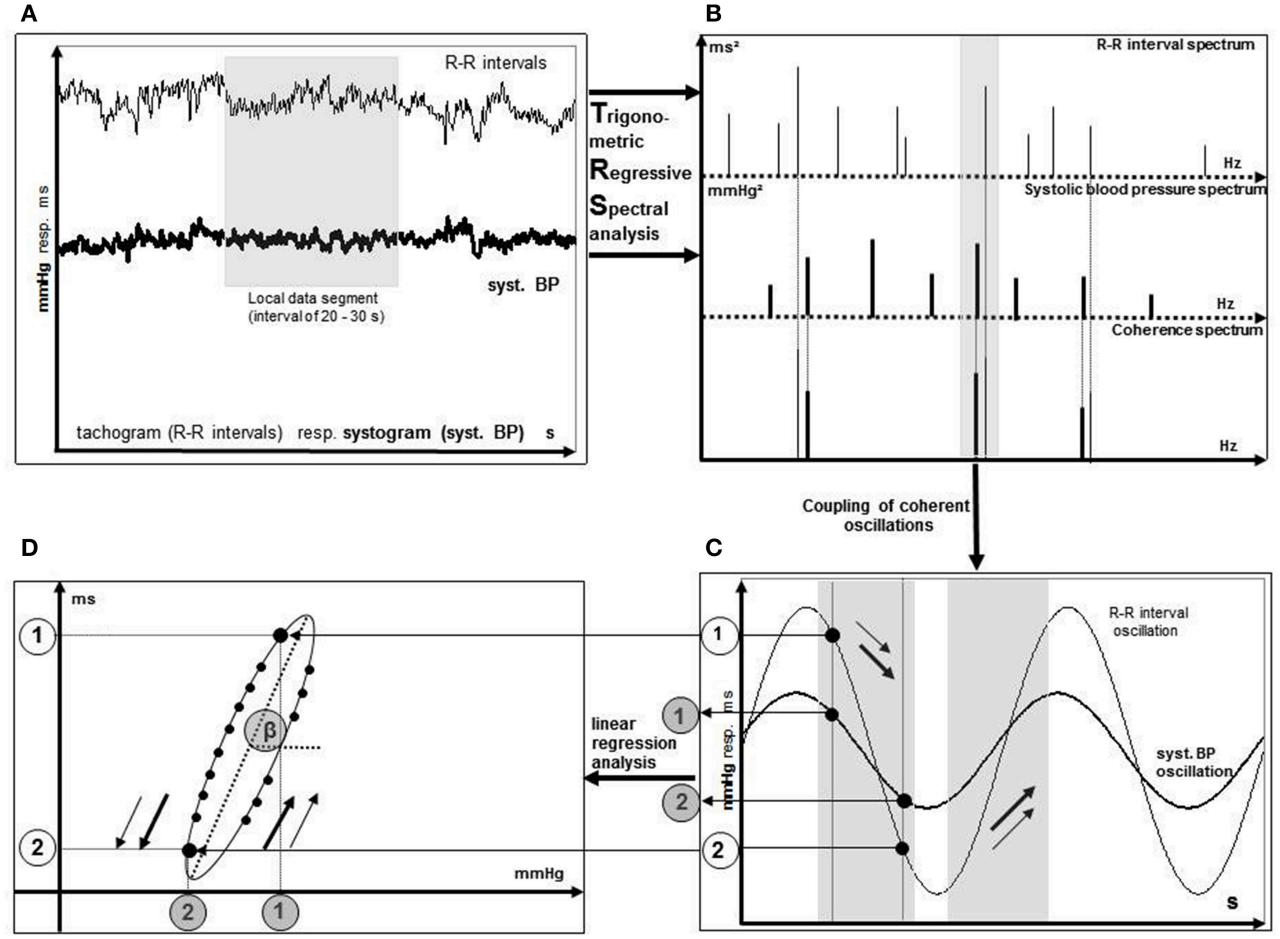
Illustration of the BRS calculation process using TRS. Spontaneous oscillations of RR intervals and systolic blood pressure **(A)** are replaced by theoretical TRS oscillations **(B)**. Calculation of BRS as the slope of the regression line **(D)** originates from coherent oscillation pairs of RR interval and systolic blood pressure **(C)**. Points 1 and 2 are two examples of coherent oscillation pairs of RR intervals and systolic blood pressures. [Taken from Gasch et al. (2011)].

**Figure 2:**
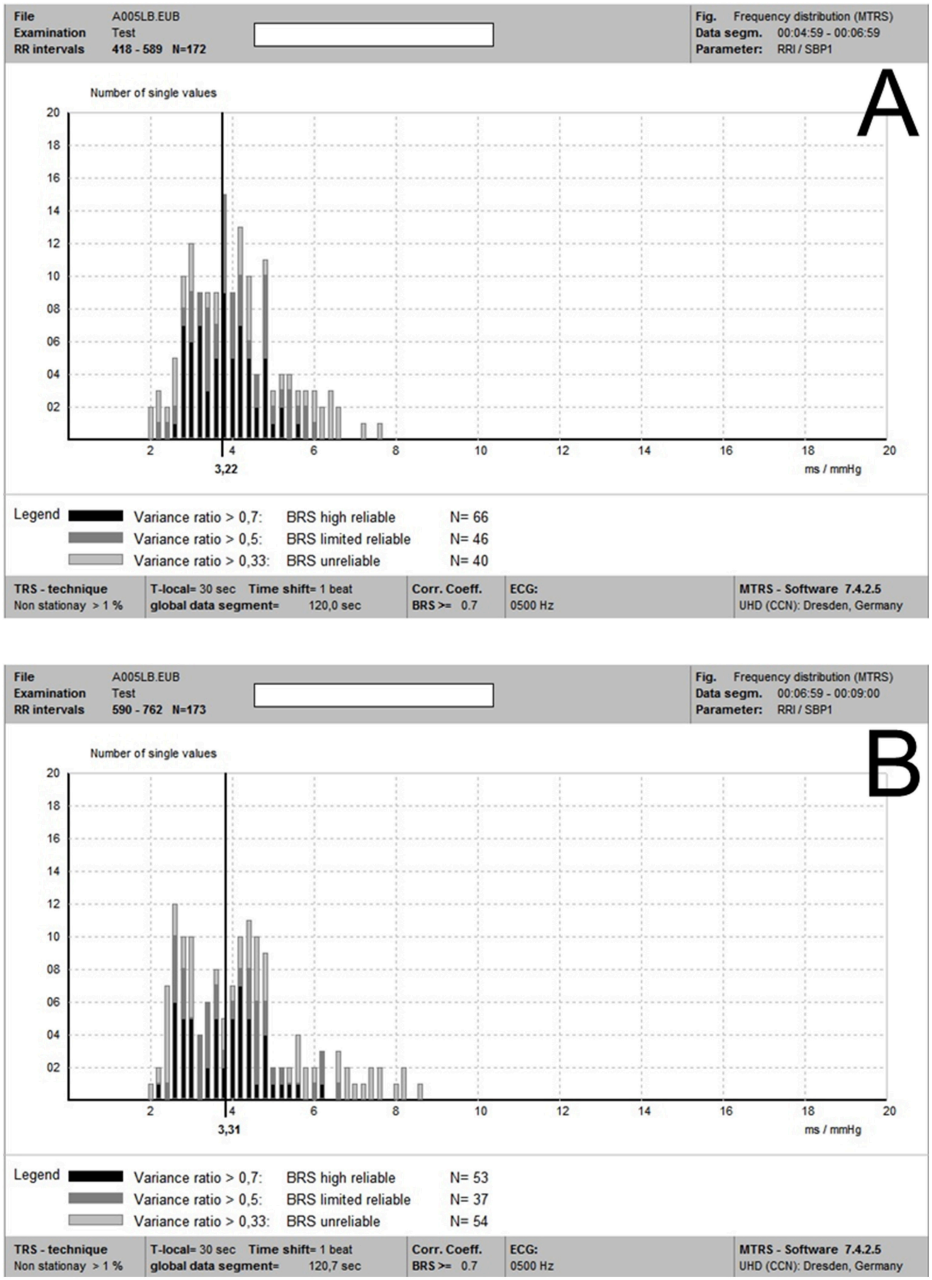
**(A**,**B)** Individual BRS-values within two different 2-min global data segments (2a and 2b, respectively) in the same recording. It is noted that although there was some degree of variability of individual local BRS-values between these two global data segments, these two mean global BRS-values were quite close.

As mentioned above, the MTRS technique uses the oscillations of SBPs and RRIs instead of their original values which are used in the sequence methods. Therefore, coherent pairs of SBP and RRI oscillations are identified, which can be correlated with one another (Gasch et al., 2011; Ziemssen et al., 2013) (Figure 1). The frequency distribution of individual BRS-values within two different 2-min global data segments are shown in Figure 2. The coherence of RRI and SBP oscillation pairs was determined by their frequencies and phase shifts. Using the TRS technique, oscillation pairs are considered coherent when the frequency difference is ≤0.025 Hz.

Let V_SYS (i) the variance reduction of the i-th coherent systolic BP oscillation and V_RR (i) be the variance reduction of the i-th coherent RR interval oscillation, then a variance ratio can be defined according to the following equations:

$$\begin{array}{l} \text{Ratio}(\text{i})=\text{V}\_\text{RR}(\text{i})\text{/V}\_\text{SYS}(\text{i})\text{ for V}\_\text{RR}(\text{i})<=\text{V}\_\text{SYS }(\text{i})\text{ or}\\ \text{Ratio}(\text{i})=\text{V}\_\text{SYS}(\text{i})\text{/V}\_\text{RR }(\text{i})\text{ for V}\_\text{RRr }(\text{i})>\text{V}\_\text{SYS }(\text{i}) \end{array}$$

This ratio can be defined for each coherent oscillation pair (i), 0 <Ratio <= 1. With all the oscillation ratios, BRS can be determined.

During the shift of the local data segments by one, two or more beats, these coherent oscillation pairs change in frequency and amplitude. Therefore, with a local data segment of 25 s, all oscillations other than the VLF band are contained at least once, and the number of individual values can be significantly increased by shifting this small data segment over a global data segment of one or more minutes.

This MTRS technique has been further developed and improved after its application in the EuroBaVar study (Ziemssen et al., 2008). In the calculations of BRS in the EuroBaVar study, all individual values have been arithmetically averaged; a weighted mean is now determined according to the following relationship:

$$\text{BRS}\_\text{opt}=(\sum \text{BRS}{\left(\text{i}\right)}^{*}\text{V}\_\text{ratio}{\left(\text{i}\right)}^{2})\text{/}\sum \text{V}\_\text{ratio}{\left(\text{i}\right)}^{2}$$

With the squaring function, an even stronger weight is placed on values close to V_ratio = 1. This reduces the influence of inconsistent BP and RR interval fluctuations (such as small BP fluctuations corresponding with large RR interval fluctuations) on the BRS calculation, because this phenomenon apparently does not reflect a baroreflex.

### BRS analyses using varying local and global data segments:

We used different local and global data segment settings for each recording. To assess the influence of different local data segment lengths, we calculated the BRS of a common 1-min global ECG and BP segment (1a) using local data segments of 12, 20, and 30 s, respectively. To explore the influence of the length of global data segments, we additionally computed BRS from a 2-min global data segment (2a) which extended 1 min from the aforementioned 1-min global data segment (1a) (the 1-min segment 1a was included in the 2-min segment 2a). To test the stability of BRS analysis using MTRS, we calculated BRS from another 2-min ECG and BP data segment (2b), which had no overlap with the other two global data segments (1a and 2a). We used a common length (30 s) of local data segments when comparing BRS-values computed from different global data segments (1a, 2a, and 2b).

### Statistical analysis

All statistical analyses were performed using SPSS for Windows (Version 23.0. Armonk, NY: IBM Corp). Data are presented as mean ± standard deviation unless stated otherwise. The Kolmogorov–Smirnov test was used to evaluate data normality. Repeated measures ANOVA or Friedman's test was employed to test differences between BRS-values obtained with different settings of data segments.

To compare the BRS estimates from different local and global data segments, we performed Spearman correlation and the Bland-Altman plot. The differences between the pairs of measurements (e.g., BRS_2a_−BRS_2b_) on the vertical axis were plotted against the means of each pair [e.g., (BRS_2a_+BRS_2b_)/2]. The Bland-Altman analysis requires that the differences should be normally distributed. Logarithmic (ln) transformation of the original BRS data was used if the differences of the BRS-values were not normally distributed and the logarithmic transformation solved this problem. To determine the potential proportional bias, we performed the Spearman correlation between the differences of the pairs and their means. We also calculated the 95% confidence intervals of the differences (also called limits of agreement) (Bland and Altman, 1986; Giavarina, 2015). *P* ≤ 0.05 were considered statistically significant.

## Results

### Comparisons between BRS obtained using different local and global data segments

Friedman's test was used for comparisons between different data segment settings. The mean BRS-values of different global data segments (using a common local data segment length of 30 s) were 10.44 ± 8.83, 11.58 ± 11.79, and 11.07 ± 11.34 ms/mmHg for 1a, 2a, and 2b in the supine position, and 5.82 ± 3.13, 5.65 ± 3.09, and 5.39 ± 3.57 ms/mmHg in the standing position, respectively. There was no significant difference between the BRS-values obtained using different global data segments.

The mean BRS-values calculated with different local data segment lengths (using the common global data segment 1a) were 12.84 ± 11.42, 11.27 ± 9.24, and 10.44 ± 8.83 ms/mmHg for local data segment lengths of 12, 20, and 30 s in the supine position, and 6.01 ± 3.56, 5.84 ± 3.31, and 5.82 ± 3.13 ms/mmHg in the standing position, respectively. There were significant differences between 12 s and the other two local data segments in the supine position (*p* = 0.013 for 12 s vs. 20 s and *p* < 0.001 for 12 s vs. 30 s), while BRS-values obtained with local data segments of 20 and 30 s were similar. The BRS-values obtained in the standing position were similar across different local data segment settings.

### Correlation analyses between BRS-values obtained using different data segment settings

There were significant correlations between all the BRS calculated using different global data segments and BRS calculated using different local data segment lengths, and all the *p*-values were <0.001. The correlation coefficients are shown in Table 1.

**Table 1:** Spearman's correlation coefficients between different BRS-values.

	**G1a**						**L30**					
	**Supine**			**Standing**			**Supine**			**Standing**		
	L12 vs. L20	L12 vs. L30	L20 vs. L30	L12 vs. L20	L12 vs. L30	L20 vs. L30	G1a vs. G2a	G1a vs. G2b	G2a vs. G2b	G1a vs. G2a	G1a vs. G2b	G2a vs. G2b
Rho	0.98	0.95	0.97	0.95	0.89	0.93	0.97	0.88	0.92	0.93	0.91	0.87

*G1a, BRS calculated using the global data segment 1a*.

*G2a, BRS calculated using the global data segment 2a*.

*G2b, BRS calculated using the global data segment 2b*.

*L12, BRS calculated using the local data segments of 12s*.

*L20, BRS calculated using the local data segments of 20s*.

*L30, BRS calculated using the local data segments of 30s*.

### Bland-altman analyses of the BRS-values obtained using different parameters

**Figure 3:**
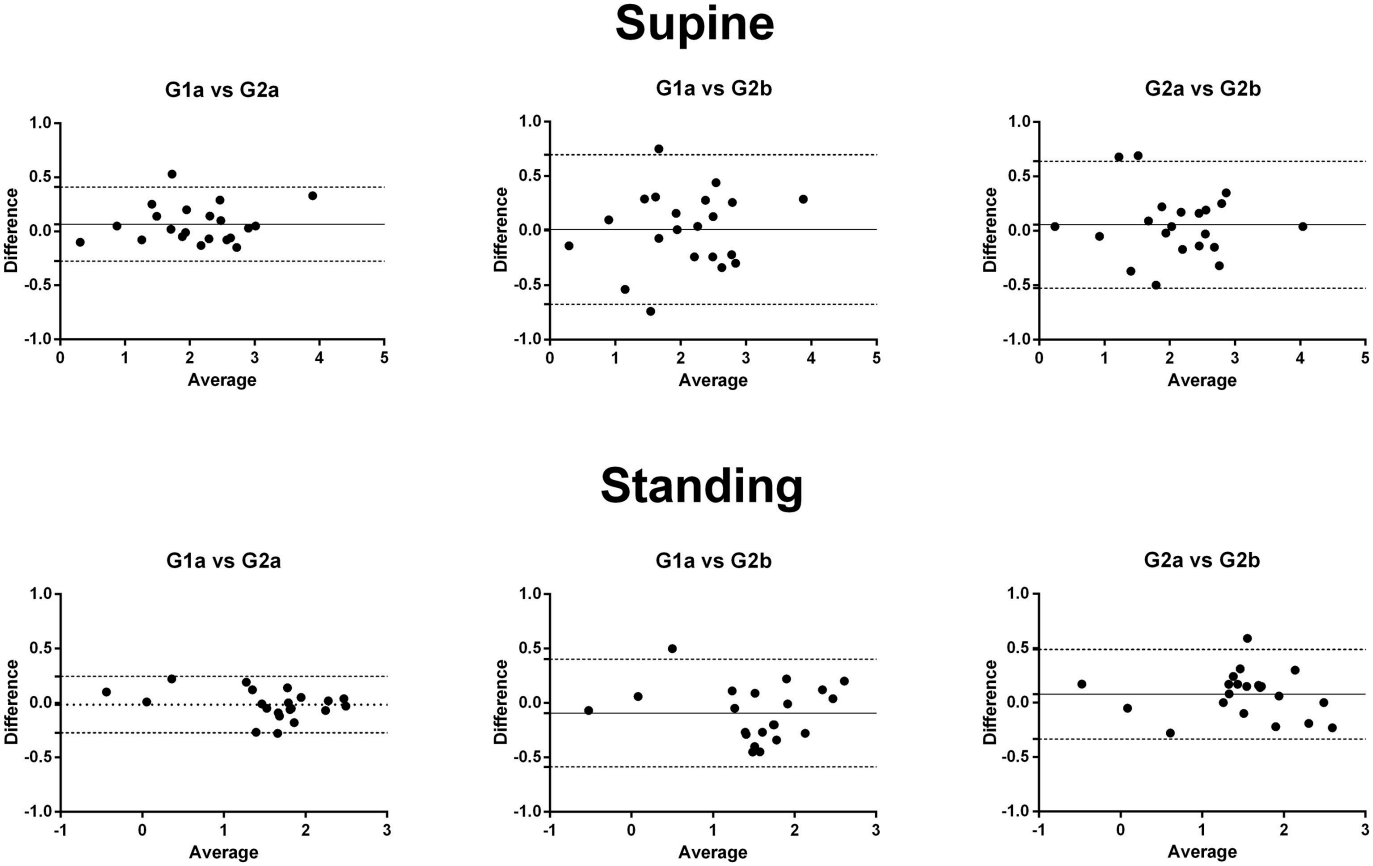
The Bland-Altman plot of the differences and means of the logarithmic transformed BRS-values using different global data segments. There was no significant fixed or proportional bias. G1a, BRS calculated using the global data segment 1a; G2a, BRS calculated using the global data segment 2a; G2b, BRS calculated using the global data segment 2b.

Comparing the BRS-values calculated using different global data segments showed no fixed bias or proportional bias, either in the supine or standing position. In addition, most of the differences between these BRS-values were relatively small regarding the corresponding mean values (Figure 3).

**Figure 4:**
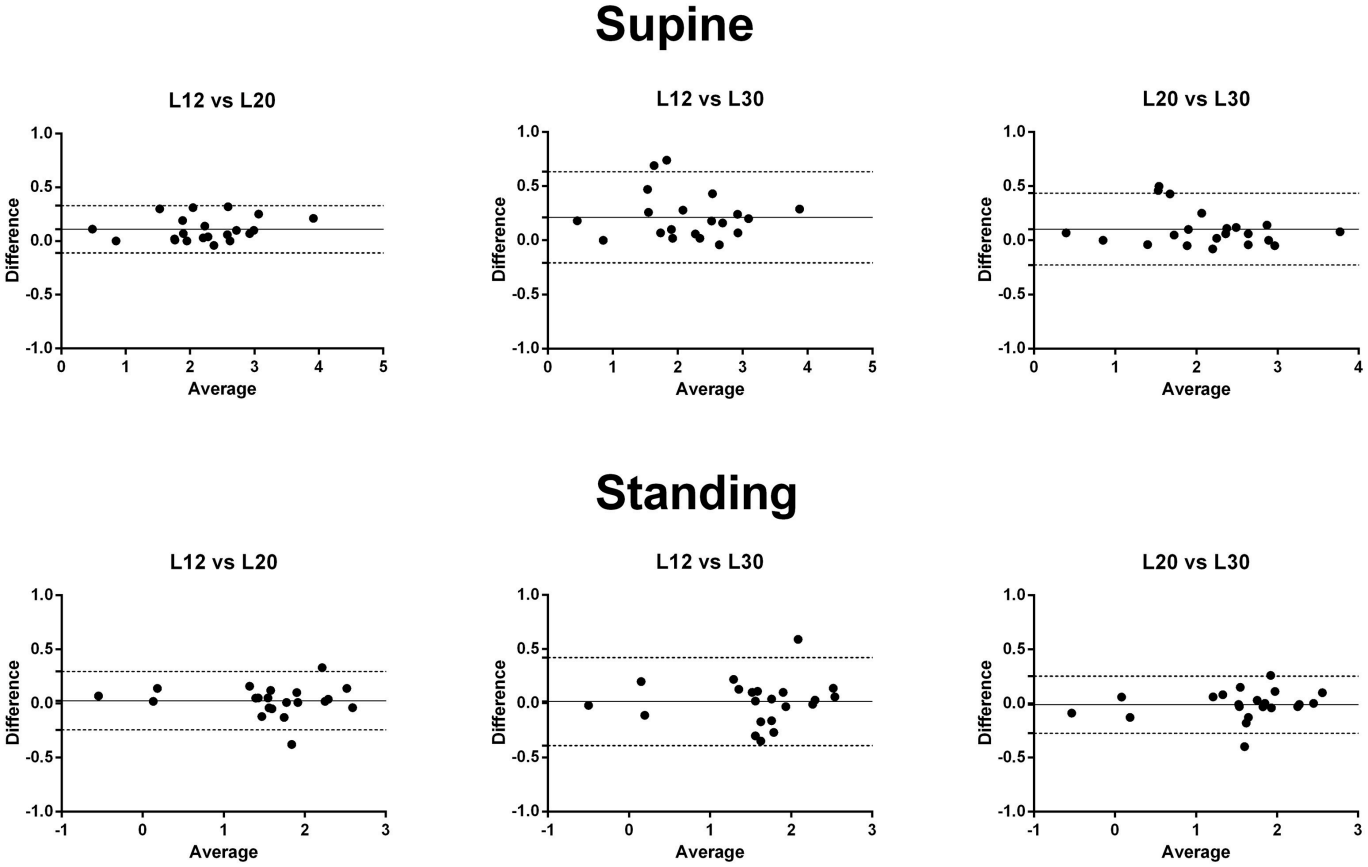
The Bland-Altman plot of the differences and means of the logarithmic transformed BRS-values using different lengths of local data segments. BRS-values obtained using local data segments of 12 s were higher than those using local data segments of 20 and 30 s (fixed bias). There was no significant proportional bias. L12, BRS calculated using the local data segments of 12 s; L20, BRS calculated using the local data segments of 20 s; L30, BRS calculated using the local data segments of 30 s.

However, when calculating BRS using the common 1a global data segment in the supine position, the BRS computed using the local data segment of 12 s was higher than those using local data segments of 20 and 30 s (fixed bias). BRS estimations using local data segments of 20 and 30 s in the supine position were similar. There was no significant fixed bias comparing BRS calculated by different local data segments in the standing position. No proportional bias was found when comparing BRS using different local data segments in the supine or standing position (Figure 4).

## Discussion

This is the first study evaluating the influences of local and global data segment settings on BRS analysis by MTRS. We found very close correlations between BRS-values calculated using different global and local data segments. Both in the supine and standing positions, using different global data segments did not significantly affect the BRS estimation. However, in the supine position, local data segments that are too short, such as 12 s, lead to overestimation of BRS.

MTRS solves several shortcomings of the widely used sequence method and spectral analysis using fast Fourier transform. The sequence method requires consecutive concordant changes of BP and heart rate, thus sometimes not enough BP and heart rate pairs can be obtained for accurate BRS calculation. In the EuroBaVar study, a large proportion of the methods using the sequence approach failed to detect autonomic failure (Laude et al., 2004). The fast Fourier transform based spectral analysis requires interpolation between real RRIs which affect the accuracy of the BRS assessment, and demands a long stationary data segment of at least 5 min which restrict its usage in dynamic processes (Rüdiger et al., 1999; Ziemssen et al., 2013). MTRS has overcome these problems using a trigonometric regression and short local data segments.

On one hand, using the coherent RRI and SBP oscillations determined by TRS other than the original RRI- and SBP-values (like in the sequence methods) could substantially increase the number of single BRS-values per time. This substitution could improve the statistical power and validity of BRS estimation (Ziemssen et al., 2013). On the other hand, the algorithm of TRS analysis provides a pure physiological spectrum using trigonometric regression in contrast to a mathematical spectrum by using the Fast Fourier transform, thus avoiding interpolation and promote the accuracy of BRS evaluation (Ziemssen et al., 2013). Figure 2 presents an illustration of the BRS analysis of two separate global data segments. Figure 2A shows the frequency distribution of individual BRS-values in a data segment of 2 min (2a) of the recording of a subject in the supine position. There are plenty of individual BRS-values detected, and the values with the greatest reliability (variance ratio ≥ 0.7) concentrated around the vertical line of the value 3.22 ms/mmHg. Figure 2B shows the individual BRS-values' distribution of another global data segment of 2min (2b) in the same recording. The averaged “optimal” BRS estimate was 3.31 ms/mmHg, which was quite close to that of the global data segment 2a. Furthermore, as mentioned in the method section, our improved MTRS analysis (after the EuroBaVar study) gives more weight on the “real” coherent SBP and RRI pairs for BRS estimation. This improvement reduces the influence of non-baroreflex mediated random fluctuations of SBP and RRI on BRS analysis. This upgrade enhances the reliability and stability of BRS analysis by MTRS, and may be part of the reason of the high consistency of BRS estimations using different global data segments.

In our study, supine BRS analysis using local data segments of 12 s produced significantly higher BRS-values compared with those using local data segments of 20 and 30 s. Because the longest LF oscillation has a wavelength of 25 s, 12 s might be too short to accurately assess the oscillations in the LF band. Increasing the local data segment length to 20 s would solve this problem and obtain similar results as those using local data segments of 30 s. Therefore, for MTRS, a local data segment length of 20–30 s is considered to be optimal for BRS analysis in the supine position. In a local data segment of TRS analysis, the oscillations must also remain constant similar to the fast Fourier transform. However, fast Fourier transform requires a stationary segment with the length of at least 5 min. For TRS, a stable local segment length of only 20–30 s seems to be enough. Therefore, MTRS can be utilized in BRS analysis during a dynamic process. Wright and colleagues conducted an analysis with a global data segment of 30 s and local data segments of 20 s, which is a good example of MTRS for a relatively short cardiovascular recording (Wright et al., 2009). In contrast to the BRS in the supine position, BRS-values applying different lengths of local data segments did not differ significantly in the standing position. The reason might be that BRS decreased during orthostasis (Friedrich et al., 2010; Reimann et al., 2010), and the difference also shrank to be non-significant.

A limitation of the MTRS analysis on BRS is that it could not distinguish the two directions of interactions between heart rate and SBP. Heart rate and BP can influence each other, thus form a closed loop. The change of BP would regulate heart rate through baroreflex (a feedback process), while heart rate also affects BP via the Frank–Starling mechanism and the runoff phenomenon (a feedforward non-baroreflex process) (Baselli et al., 1988; Taylor and Eckberg, 1996; Javorka et al., 2017). Recent studies have shown that causal analyses could discriminate the effect of SBP on heart rate (the feedback process, mediated by baroreflex) from the effect of heart rate to SBP (the feedforward process) (Porta et al., 2011, 2013; Svacinova et al., 2015; Javorka et al., 2017). Although MTRS based BRS analysis is a non-causal analysis, it showed good performance in the EuroBaVar study and agreed well with the modified Oxford method which was viewed as the gold standard of BRS estimation (Gasch et al., 2011). Future studies comparing causal analysis, MTRS and modified Oxford method would be helpful to clarify this issue.

In conclusion, BRS estimation by MTRS using different global data segments could acquire highly consistent results. However, too short local data segments such as 12 s would overestimate BRS in the supine position, and the optimal lengths of local data segments should be 20–30 s.

## Author contributions

Conception and design of the study: TZ, HR. Acquisition, analysis and interpretation of data: KL, HR, and RH. Drafting the manuscript: KL, TZ. All the other authors critically revised the draft and approved the final version.

### Conflict of interest statement

The authors declare that the research was conducted in the absence of any commercial or financial relationships that could be construed as a potential conflict of interest.
